# Academic general practice/family medicine in times of COVID-19 – Perspective of WONCA Europe

**DOI:** 10.1080/13814788.2020.1855136

**Published:** 2020-12-18

**Authors:** Adam Windak, Thomas Frese, Eva Hummers, Zalika Klemenc Ketis, Sonia Tsukagoshi, Josep Vilaseca, Shlomo Vinker, Mehmet Ungan

**Affiliations:** a Department of Family Medicine, Jagiellonian University Medical College, Krakow, Poland; b Institute of General Practice & Family Medicine, Medical Faculty, Martin-Luther-University Halle-Wittenberg, Halle, Germany;; c Department of General Practice, Georg-August-Universitat Gottingen, Gottingen, Germany; d Community Health Centre Ljubljana, Slovenia;; e Department of Family Medicine, Faculty of Medicine, University of Maribor, Slovenia;; f Department of Family Medicine, Faculty of Medicine, University of Ljubljana, Ljubljana, Slovenia;; g Royal College of General Practitioners, London, UK;; h Consorci d'Atenció Primàrìa Barcelona Esquerra, Barcelona, Spain;; i Universitat de Barcelona, Barcelona, Spain;; J Department of Family Medicine, Sackler Faculty of Medicine, Tel Aviv University, Tel Aviv, Israel; k Department of Family Medicine, Ankara University School of Medicine, Ankara, Turkey

**Keywords:** Infectious diseases, quality of care, public health and community medicine, health care organisation and management, medical education

## Abstract

COVID-19 outbreak has significantly changed all aspects of general practice in Europe. This article focuses on the academic challenges for the discipline, mainly in the field of education, research, and quality assurance. The efforts of the European Region of the World Organisation of National Colleges, Academies, and Academic Associations of General Practitioners/Family Physicians (WONCA Europe) to support academic sustainability of the discipline in the time of pandemic are presented. Medical education was affected by the pandemic, threatening both its productivity and quality. Emerging new educational methods might be promising, but the results of their rapid implementation remain uncertain. A relatively small number of publications related to COVID-19 and general practice is available in the medical literature. There is a shortage of original data from general practice settings. This contrasts with the crucial role of GPs in fighting a pandemic. COVID-19 outbreak has opened widely new research areas, which should be explored by GPs. Maintaining the quality of care and safety of all patients during the COVID-19 pandemic is the utmost priority. Many of them suffer from poor access or inadequate management of their problems. Rapid implementation of telemedicine brought both threats and opportunities. The COVID-19 pandemic also challenged doctors’ safety and well-being. These aspects will require discussion and remedy to prevent deterioration of the quality of primary care. WONCA Europe is making a multi-faceted effort to support GPs in difficult times of the pandemic. It is ready to support future efforts to uphold the integrity of family medicine as an academic discipline.

KEY MESSAGES

- Both medical education and research in the field of general practice/family medicine have been challenged by the COVID-19 pandemic, opening new perspectives in these areas.
- COVID-19 has significantly caused a rapid and successful implementation of the telemedicine and remote care model, however, the long-term consequences of these changes remain uncertain.
- WONCA Europe makes efforts to uphold the integrity of family medicine as an academic discipline, threatened by the COVID-19 pandemic.

## Introduction

The SARS-COV-2 infection has hit almost all countries of the world in an unprecedented manner. In just a few days, the functioning of individuals and entire societies changed drastically, and their health and lives were seriously threatened. Nowadays, health care systems have been the focus of attention all over the world. Many eyes are directed on family doctors, who stand on the front line of the fight against the virus. At the beginning of the outbreak, many of them paid the highest sacrifice with their own lives in the fight against the global pandemic.

Several articles have been published on various clinical aspects of COVID-19. In this background article, we want to discuss the risks and challenges that the current pandemic presents to academic aspects of General Practice/Family Medicine in Europe. Precisely, we want to focus on the challenges in the field of education, research, and quality assurance – three main academic pillars of our discipline. Moreover, we want to present an overview of the efforts undertaken by the European Region of the World Organisation of National Colleges, Academies, and Academic Associations of General Practitioners/Family Physicians (WONCA Europe) to support the Primary Health Care doctors in their fight against the pandemic.

## Medical education

The COVID-19 pandemic has also significantly affected teaching in all levels of medical education. Most medical schools have suspended regular classes for students, moving them from classrooms to the internet. Overnight, virtual-learning, video-conferencing, social media contacts, and broadly understood telemedicine have become substitutes for traditional medical education [1]. In fear of spreading the virus, rotations in wards and outpatient clinics were also stopped, limiting the possibility for students and young doctors to gain knowledge and broaden competencies through direct contact with real patients. Such decisions are primarily dictated by the fear that frequent rotations of students and residents will cause them to become potential infection vectors [2]. In many places, however, both students and residents joined the fight against pandemics, acquiring new competencies that may prove extremely useful in the future.

Suspending clinical classes or replacing them with virtual teaching raises concerns about the quality of education and the competencies of graduates [3]. On the other hand, however, significant delays in the didactic process, especially in the case of students and residents of final years of training, may result in a delay of subsequent groups of doctors in entering the health care system, which in many countries in Europe may significantly aggravate already large shortages of medical staff.

The introduction of blended medical education, which has been postulated for many years, has accelerated rapidly during the pandemic with a visible shift of focus to forms of distance learning. The necessity of virtual education poses new challenges not only for students but also and perhaps above all, for teachers [4]. Some of them, especially those belonging to the older generation and teaching clinical competence in the GP practice, have significant difficulties in conducting distance learning. An important challenge is also the need to reconcile the organisation of reliable and credible exams with the need to maintain social distance. The immediate stoppage of traditional medical education in many places was accompanied by the sudden appearance of new solutions in this area. Their nature, scope, and consequences are currently completely unknown and are the subject of ongoing research [5].

## Research agenda

During the initial six months of the COVID-19 pandemic, approximately 18,000 publications related to the topic were listed on PubMed [6]. A quick search based on keywords revealed a minimal number of around 170 publications associated with COVID-19 and general practice. Most are position papers, recommendations, or non-systematic reviews. The main topics which are covered by the current published literature are: (i) telemedicine and remote care [7], (ii) monitoring and self-assessment of possible symptoms [8], (iii) medical education [9], (iv) training to deal with COVID-19 in the practice [10], (v) the current situation in general practice [11], (vi) how general practice is expected to change during and after the COVID-19 pandemic [12] and (vii) the burden COVID-19 brings to health care providers [13], but general practitioners were not well represented in most of the surveys assessing this [14]. Also, papers describing COVID-19 cases’ characteristics from outpatient settings are scarce [15].

Due to the dynamic situation during the last months and the relatively short time after the onset of the pandemic, there is a lack of original data, especially from general practice settings. This contrasts with the vital role of general practitioners and the needed resources at the frontline of contact with possibly COVID-19 positive persons [16].

New public health challenges also present a different area of research, for example, the tracing and testing of persons with COVID-19 contact, assessing the effects of lockdown measures and their loosening, but also researching and potentially amending their adverse effects, for example, on mental health, violence in households/families, or on economic safety and prosperity [17].

Specific research questions arise from the current state and the current lack of knowledge and need the contribution of GPs to be answered:

- What is the burden of COVID-19 for general practitioners and their teams?
- What are the strategies to protect general practitioners and their teams effectively and efficiently?
- What could future strategies for COVID-19 testing be?
- Which were and are the direct and indirect effects of COVID-19 on morbidity and mortality?
- What approaches could GPs take to cover up with the unmet health needs?
- What was the effectiveness of measurements taken to control COVID-19?
- What could be a possible long-term strategy to deal with COVD-19 in populations?
- How to deal with possible COVID-19 infections during phases of high upper respiratory tract infections (URTI) incidence due to a broader spectrum of viruses?

Since it is only possible to work on some aspects retrospectively, it is now the time to conceptualise and plan the investigations. The gained knowledge may be helpful in future waves of the COVID-19 pandemic or even other epidemics.

## Quality of care

Maintaining the quality of care and safe management of all patients in primary care faced several challenges during the COVID-19 pandemic. There have been several steps in the approach used by the guidelines for family medicine: first, a patient examination by family doctors was disapproved. Family doctors used phone interviews to assess their patients and advised them to stay at home [18]. In severe cases, patients were referred to the hospital. Some weeks later, the second step recommended that family doctors examine patients without any testing available. At this stage, family doctors learned about the main features of COVID-19 through direct observation. In the third stage, blood tests and X-rays were used to assess the severity of the patients’ conditions to decide better which patients need a referral to the hospital. Most recently, point-of-care testing with polymerase chain reaction (PCR) test has been used. These rapidly changing tasks assigned to GPs at different stages of the fight against a pandemic challenge the quality of their care.

As a result of the need to halt the spreading of the disease and protect healthcare workers, most primary care patients were consulted remotely. The COVID-19 pandemic transformed healthcare systems worldwide, with telemedicine, or virtual healthcare, being one of the key revolutions [19]. Providers with an existing telehealth infrastructure experienced slow implementation of new technologies in the past. During the recent pandemic, rapid adoption of telemedicine by both patients and providers occurred. Obstacles like reimbursement issues, lack of comfort with telemedicine technologies of patients and doctors, and a perceived need for telemedicine limited to remote rural areas have been turned around in the face of the need for social distancing and quarantine as a way to stop the pandemic. Options for telemedicine are diverse and may include written, audio or video communication between the patient and the family physician. Written communication may consist of sharing documents or even photos in a secured transferring system. Traditional telephone consultations were used by family doctors long before the COVID-19 pandemic, but modern ‘remote’ medicine is conducted through a secure video meeting along with the use of advanced remote technology for physical examination and vital signs monitoring. Digital remote examination tools, such as the digital stethoscope and otoscope, have been incorporated into clinical practice and maybe complementary for video telemedicine solutions [20].

The use of telehealth as ‘forward triage’ – the sorting of patients before they arrive at the practice allows patients to be efficiently screened for COVID-19 [21], but the opportunity for physicians and patients to communicate 24 h a day, using smartphones or webcam-enabled computers challenges the classic role of the tight long-term doctor–patient relationship – one of the pillars of family medicine. COVID-19 accelerating the adoption of telehealth may improve health care access in remote areas, but it may also reduce the motivation to invest significant resources in rural infrastructure [22].

Previous studies suggest that phone consultations can be used in some situations (i.e. follow-up) [23], but have many safety issues (i.e. difficult communication, absence of physical examination, lack of comprehensive approach) [24]; E-consultations and video consultations may improve some aspects of care delivery, but improvements need to be done to manage the safety of such consultations [25].

Remote consultations resulted in remote prescribing. Here, the danger of both over- and deprescribing emerged. In ordinary times, deprescribing should be considered when the potential for harm outweighs the benefit of medicine [26]. However, there is currently not enough evidence to support wholesale changes to patients’ medication, since such action requires careful assessment, follow-up, and safety-netting [27]. Overprescribing for example of antibiotics, analgesics, and benzodiazepines might occur due to new restrictions in the number of face-to-face consultations performed.

The ongoing pandemic also threatened equity in health care. Certain vulnerable populations (i.e. people with disabilities, or people with insecure housing conditions) may be impacted more significantly by COVID-19. This can be mitigated if simple actions and protective measures are taken by key stakeholders [28]. Public health and social measures must be tailored to local structures, conditions, and epidemiology. Another potential threat is the underserviced patients with problems not related to COVID-19. While most attention was given to potential COVID-19 patients, other patients requiring care were at risk of being left behind, seriously questioning the equity and safety of care. Therefore, maintenance of regular services for the entire population of patients cared for by GPs should be one of the critical priorities [29].

## WONCA Europe initiatives

During the pandemic, WONCA Europe engaged in several different areas: the spread of information, education, research, and cooperation/visibility.

WONCA Europe wanted to keep the flow of information to its members and this was ensured by establishing the COVID-19 resource page at the beginning of March 2020 [30]. The information is targeted for use by general practitioners/family physicians in Europe. WONCA Europe also recommended prioritising guidance from the country’s specific local health authorities. A presidential letter on the pandemic was sent to all member organisations [31]. Additionally, information was spread through a newly established newsletter, with current topics sent to all member organisations. World Patients Safety day was celebrated by WONCA Europe and Association for quality and safety in family medicine (EQuiP) by conveying the message on this year’s topic (workers’ health) and a short video on patient safety [32]. Several other activities were also delivered to spread the news, such as an interview with the president, a video message by the president on COVID-19, and professional articles [33].

WONCA Europe also aimed to offer education on various topics associated with the pandemic. Several webinars were held on primary care during COVID-19. Most were organised jointly between WONCA Europe and other organisations or networks, such as WHO, the European Forum for Primary Care (EFPC) and others.

As there is a lack of evidence on primary care during the pandemic, WONCA Europe and its networks also engaged in research. EURACT investigated education experiences during the pandemic. Currently, a large study on quality and safety during the pandemic is underway, with the cooperation between Ghent University and EQuiP.

WONCA Europe put efforts into cooperation with key stakeholders and its visibility with outside member organisations. It signed and realised the statement on quality and safety (with EQuiP), on COVID pandemic with EFPC and with Vasco da Gama, and on digital health and telemedicine for primary care with the WHO Regional Committee for Europe. A meeting was held with WHO Europe on the topic of family physicians and the pandemic: a way forward, trying to draw common WHO/WONCA Europe conclusions and suggestions for the future are goals. Meetings were also held with the European Medicines Agency (EMA), the European Union of General Practitioners (UEMO), and the European Cancer Organisation (ECCO), to cope with the future challenges in different areas. WONCA Europe also started a process to identify the core values of family medicine. A special working group is assessing available document, discussing the possible effects that changing communities and the changing world are having on the core values of the discipline, and developing a list of core values. More information about WONCA Europe initiatives related to the academic aspects of General Practice/Family Medicine in relation to the ongoing pandemic is available on the WONCA Europe website [33].

## Future challenges for the academic community

Future challenges for the academic community are associated with three main pillars of the discipline of family medicine: education, research, and quality and safety of clinical work.

Education in family medicine in Europe is mostly performed according to six main competencies of family medicine and incorporated into the EURACT Educational Agenda for family medicine/general practice [34]. The times of the pandemic are significantly changing the content and methods of teaching. Therefore, the content of teaching should be adapted to the needs of family physicians and new methods of teaching (such as online lectures and online examinations) should be evaluated/validated for their use in teaching. Teachers must be taught about these changes to gain new competencies to cope with all the changes adequately. All these measures will also assure the quality of teaching in the future.

Currently, there is a lack of evidence from primary care regarding not only the management of COVID-19 patients but also regarding the management of all other patients. Therefore, studies are needed to gain evidence, which will enable writing evidence-based guidelines, ultimately leading to quality and safety in the family medicine practice. A challenge here is gaining the required finances for research. Here, the academic community should connect with important stakeholders to acquire enough resources for performing high-quality research involving all European countries.

In the clinical area, the challenges are how to ensure high-quality care for all patients, taking into account the rise of remote consultations, the assurance of the safety of health workers and patients, and the lack of healthcare workers that was evident even before the pandemic. New models of work will emerge and it will be the role of the academic community to direct and prioritise the usefulness of the new organisational models. Professionals from different fields of health care will need to cooperate to ensure the most efficient interdisciplinary approach.

Health workers’ safety and well-being is another challenge. Effective models of coping with anxiety, stress, fear for their own safety, health, and the lives of their relatives and patients must be developed. Physician burnout is well-documented in the literature and is marked by a feeling of a lack of agency, detachment, and disillusionment and can lead to irritability, poor decisions, and have negative impacts on interpersonal relationships [18]. Untreated and unmanaged conditions can lead to mental health issues, including depression and substance misuse for the doctor.

Psychological threats might be especially dangerous for residents and other junior physicians. Family doctors early in their career need to be especially careful, as they are often given more workload than seniors and are still developing their own coping strategies [35]. Care ethics specify that to avoid burnout, we need to foster an environment where there is time to stop and reflect to understand our own boundaries and limits [36].

## Conclusion

The pandemic has challenged medical education, research, and quality assurance in General Practice/Family Medicine. These areas have to adapt to the new needs and requirements that emerged in times of COVID-19. The pandemic can serve as an opportunity to scale-up primary care, mainly in the areas of interprofessional care, remote consultations, and empowering people for self-care, thus achieving a higher quality of care, reducing the workload on the physicians, and reducing health care expenses. It has also highlighted the importance of physician well-being and we must continue the discussion on ‘safe staffing, safe resources and safe models of care’ long after the pandemic. Family doctors across Europe are facing new clinical challenges and have to adapt their practices to the unique circumstances created by COVID-19. Academic general practice has to follow and support these changes. WONCA Europe and all its network organisations are ready to join and lead this process.

## References

[1] Dedeilia A, Sotiropoulos MG, Hanrahan JG, et al. Medical and surgical education challenges and innovations in the COVID-19 era: a systematic review. In Vivo. 2020;34(3):1603–1611. 32503818 10.21873/invivo.11950 PMC8378024

[2] Ahmed H, Allaf M, Elghazalya H. COVID-19 and medical education. Lancet Infect Dis. 2020;20:e79. 10.1016/S1473-3099(20)30226-7 PMC7270510 32213335

[3] Ferrel MN, Ryan JJ. The impact of COVID-19 on medical education. Cureus. 2020;12(3):e7492. 32368424 10.7759/cureus.7492 PMC7193226

[4] Sahu P. Closure of universities due to Coronavirus Disease 2019 (COVID-19): impact on education and mental health of students and academic staff. Cureus. 2020;12(4):e7541. 32377489 10.7759/cureus.7541 PMC7198094

[5] Michels NRM, Scherpbier N, Karppinen H, et al. Do you know how COVID-19 is changing general practice/family medicine education? Educ Prim Care. 2020;31(3):196–197. 32339073 10.1080/14739879.2020.1755609

[6] www.pubmed.gov. [cited 2020 June 7].

[7] Thulesius H. Increased importance of digital medicine and eHealth during the Covid-19 pandemic. Scand J Prim Health Care. 2020;38(2):105–106. 32484725 10.1080/02813432.2020.1770466 PMC8570755

[8] Ming DK, Sorawat S, Chanh HQ, et al. Continuous physiological monitoring using wearable technology to inform individual management of infectious diseases, public health and outbreak responses. Int J Infect Dis. 2020;96:648–654. 32497806 10.1016/j.ijid.2020.05.086 PMC7263257

[9] Roskvist R, Eggleton K, Goodyear-Smith F. Provision of e-learning programmes to replace undergraduate medical students’ clinical general practice attachments during COVID-19 stand-down. Educ Prim Care. 2020;31(4):247–254. 32469632 10.1080/14739879.2020.1772123

[10] Desborough J, Hall Dykgraaf S, Rankin D, et al. The importance of consistent advice during a pandemic: an analysis of Australian advice regarding personal protective equipment in healthcare settings during COVID-19. Aust J Gen Pract. 2020;49(6):369–372. 32464735 10.31128/AJGP-04-20-5374

[11] Yu EYT, Leung WLH, Wong SYS, et al. How are family doctors serving the Hong Kong community during the COVID-19 outbreak? A survey of HKCFP members. Hong Kong Med J. 2020;26:176–183. 32475841 10.12809/hkmj208606

[12] Khan N, Jones D, Grice A, et al. A brave new world: the new normal for general practice after the COVID-19 pandemic. BJGP Open. 2020;4(3):bjgpopen20X101103. 10.3399/bjgpopen20X101103 PMC7465568 32487520

[13] Chew QH, Chia FL, Ng WK, et al. Psychological and coping responses to COVID-19 amongst residents in training across ACGME-I accredited specialties in Singapore. Psychiatry Res. 2020;290:113146. 32502830 10.1016/j.psychres.2020.113146 PMC7254004

[14] Bohlken J, Schömig F, Lemke MR, et al. [COVID-19 pandemic: stress experience of healthcare workers - a short current review]. Psychiatr Prax. 2020;47(4):190–197. 32340048 10.1055/a-1159-5551 PMC7295275

[15] Cohen PA, Hall LE, John JN, et al. The early natural history of SARS-CoV-2 infection: clinical observations from an urban, ambulatory COVID-19 clinic. Mayo Clin Proc. 2020;95(6):1124–1126. 32451119 10.1016/j.mayocp.2020.04.010 PMC7167572

[16] Stedman M, Lunt M, Davies M, et al. COVID-19: generate and apply local modelled transmission and morbidity effects to provide an estimate of the variation in overall relative healthcare resource impact at general practice granularity. Int J Clin Pract. 2020;74(9):e13533. 32392377 10.1111/ijcp.13533

[17] COVID-19 and violence against women. What the health sector/system can do [Internet]. Geneva (Switzerland): World Health Organization; 2020 [cited 2020 June 8]. Available from: https://apps.who.int/iris/rest/bitstreams/1274324/retrieve

[18] Marshall M, Howe A, Howsam G, et al. COVID-19: a danger and an opportunity for the future of general practice. Version 2. Br J Gen Pract. 2020;70(695):270–271. 32393503 10.3399/bjgp20X709937 PMC7219629

[19] Mann DM, Chen J, Chunara R, et al. Nov O. COVID-19 transforms health care through telemedicine: evidence from the field. J Am Med Inform Assoc. 2020;27(7):1132–1135. 32324855 10.1093/jamia/ocaa072 PMC7188161

[20] McDaniel NL, Novicoff W, Gunnell B, et al. Comparison of a novel handheld telehealth device with stand-alone examination tools in a clinic setting. Telemed J E Health. 2019;25(12):1225–1230. 30561284 10.1089/tmj.2018.0214 PMC6918850

[21] Hollander JE, Carr BG. Virtually perfect? Telemedicine for Covid-19. N Engl J Med. 2020;382(18):1679–1681. 32160451 10.1056/NEJMp2003539

[22] Wright B, Fraher E, Holder MG, et al. Will community health centers survive COVID-19? J Rural Health. 2020;0:1–4. 10.1111/jrh.12473 PMC7276796 32415879

[23] Ansell D, Crispo JAG, Simard B, et al. Interventions to reduce wait times for primary care appointments: a systematic review. BMC Health Serv Res. 2017;17(1):295. 28427444 10.1186/s12913-017-2219-y PMC5397774

[24] van Galen LS, Car J. Telephone consultations. Br Med J. 2018;360:k1047. 29599197 10.1136/bmj.k1047

[25] Mold F, Hendy J, Lai YL, et al. Electronic consultation in primary care between providers and patients: systematic review. JMIR Med Inform. 2019;7(4):e13042. 31793888 10.2196/13042 PMC6918214

[26] Farrell B, Mangin D. Deprescribing is an essential part of good prescribing. Am Fam Physician. 2019;99(1):7–9. 30600973

[27] Phizackerley D. Deprescribing in the time of covid-19. Drug Ther Bull. 2020;58(6):82. 32354724 10.1136/dtb.2020.000027

[28] Disability considerations during the COVID-19 outbreak [Internet]. Geneva (Switzerland): World Health Organization; 2020 [cited 2020 June 8]. Available from: https://www.who.int/publications-detail/disability-considerations-during-the-covid-19-outbreak

[29] Kidd MR. Five principles for pandemic preparedness: lessons from the Australian COVID-19 primary care response. Br J Gen Pract. 2020;70(696):316–317. 32571772 10.3399/bjgp20X710765 PMC7311106

[30] COVID-19 resources for general practitioners/family physicians [Internet]. Ljubljana (Slovenia): WONCA Europe; 2020 [cited 2020 October 11]. Available from: https://www.woncaeurope.org/kb/covid-19-resources-for-general-practitioners-family-physicians

[31] Presidential letter on COVID-19 [Internet]. Ljubljana (Slovenia): WONCA Europe; 2020 [cited 2020 October 11]. Availabe from: https://www.woncaeurope.org/news/view/presidential-letter-on-covid-19

[32] Respect and gratitude to health workers on “World Patient Safety Day” [Internet]. Ljubljana (Slovenia): WONCA Europe; 2020 [cited 2020 October 11]. Availabe from: https://www.woncaeurope.org/news/view/respect-and-gratitude-to-health-workers-on-world-patient-safety-day-2020

[33] WONCA Europe is working [Internet]. Ljubljana (Slovenia): WONCA Europe; 2020 [cited 2020 October 11]. Availabe from: https://www.woncaeurope.org/file/89efe462-1b2f-4e27-998c-e419b3598c59/Oct%202020%20WONCA%20Europe%20President's%20Report.pdf

[34] Heyrman J. EURACT educational agenda, European Academy of Teachers in General Practice EURACT, Leuven 2005. Ljubljana (Slovenia): WONCA Europe; 2020 [cited 2020 October 11]. Availabe from: https://euract.woncaeurope.org/sites/euractdev/files/documents/publications/official-documents/euract-educationalagenda.pdf

[35] Azam K, Khan A, Alam MT. Causes and adverse impact of physician burnout: a systematic review. J Coll Physicians Surg Pak. 2017;27(8):495–501. 28903843

[36] Casalini B. Care of the self and subjectivity in precarious neoliberal societies. Insights Anthropol. 2019;3:134–139.

